# A comparative study of MassARRAY and GeneXpert assay in detecting rifampicin resistance in tuberculosis patients’ clinical specimens

**DOI:** 10.3389/fmicb.2024.1287806

**Published:** 2024-02-07

**Authors:** Ruixia Liang, Jiankang Li, Yue Zhao, Haoran Qi, Shengjuan Bao, Fen Wang, Hongfei Duan, Hairong Huang

**Affiliations:** ^1^Tuberculosis Department, Henan Chest Hospital, Zhengzhou, China; ^2^Clinical Laboratory, Henan Chest Hospital, Zhengzhou, China; ^3^National Clinical Laboratory on Tuberculosis, Beijing Key Laboratory for Drug-Resistant Tuberculosis Research, Beijing Chest Hospital, Beijing Tuberculosis and Thoracic Tumor Institute, Capital Medical University, Beijing, China; ^4^Tuberculosis Department, Beijing Chest Hospital, Capital Medical University, Beijing, China

**Keywords:** tuberculosis, MALDI-TOF MS, MassARRAY, Xpert assay, drug resistance, reliability

## Abstract

**Objectives:**

Matrix-assisted laser desorption ionization-time of flight mass spectrometry (MALDI-TOF MS) has emerged as a potent tool for detecting drug resistance in tuberculosis (TB); however, concerns about its reliability have been raised. In this study, we assessed the reliability of MassARRAY (Sequenom, Inc.), which is a MALDI-TOF MS-based method, by comparing it to the well-established GeneXpert assay (Cepheid) as a reference method.

**Methods:**

A retrospective study was conducted using laboratory data retrieved from Henan Chest Hospital (Zhengzhou, China). To ensure a rigorous evaluation, we adopted a comprehensive assessment approach by integrating multiple outcomes of the Xpert assay across various specimen types.

**Results:**

Among the 170 enrolled TB cases, MassARRAY demonstrated significantly higher sensitivity (85.88%, 146 of 170) compared to the Xpert assay (76.62%, 118 of 154) in TB diagnosis (*p* < 0.05). The concordance in detecting rifampicin resistance between MassARRAY and the combined outcomes of the Xpert assay was 90%, while it was 97.37% (37 of 38) among smear-positive cases and 89.06% (57 of 64) among culture-positive cases. When compared to the phenotypic susceptibility outcomes of the 12 included drugs, consistency rates of 81.8 to 93.9% were obtained, with 87.9% for multiple drug resistance (MDR) identification.

**Conclusion:**

MassARRAY demonstrates high reliability in detecting rifampicin resistance, and these findings may offer a reasonable basis for extrapolation to other drugs included in the test panel.

## Introduction

Tuberculosis (TB) remains a significant contributor to infectious disease-related mortality worldwide. In 2022, an estimated 10.6 million individuals were diagnosed with TB. Among them, the rate of multiple drug-resistant (MDR, defined as simultaneous resistance to at least isoniazid and rifampin)/rifampin-resistant (RR)-TB was 3.3% among new cases and 17% among previously treated cases (World Health Organization, 2023). TB drug resistance is broadly categorized as RR, MDR, or extensively drug-resistant (XDR). Leveraging the rapid, sensitive, and reliable features of the molecular diagnostics of TB, many more drug-resistant patients have been diagnosed timely. However, the complete drug resistance patterns exhibited by individual patients can vary widely and often go undetected. This significant detection gap is primarily caused by the shortage and limitations of available diagnostics.

Conventional phenotypic drug susceptibility testing (pDST) provides coverage for a wide range of drugs; however, the complexity associated with this technique has limited its widespread application. Additionally, the long turnaround time hampers its effectiveness in guiding the establishment of an appropriate treatment regimen. Concerns about its reliability and reproducibility further constrain its use as a diagnostic tool for drug resistance (van Deun et al., 2011). Commercial PCR-based molecular testing addresses the prolonged time delay inherent in pDST while also exhibiting reduced technical complexity. However, it has an obvious drawback in that the targeted genes, i.e., the drugs covered, are limited (World Health Organization, 2021). For instance, the Xpert MTB/RIF assay (Xpert) (Cepheid Inc., Sunnyvale, CA, USA) solely identifies rifampin resistance associated with the *rpoB* mutation. The GenoType MTBDRplus (Hain Lifescience) can only detect mutations in genes related to resistance to rifampin (*rpoB*) and isoniazid (*inhA* and *katG*) (Gu et al., 2015). Even though the Xpert MTB/XDR (Cepheid, USA) covers six drugs, this number remains insufficient (Xie et al., 2017). Without knowledge of the complete drug resistance pattern, drugs (without information on their susceptibility) must be selected without a clear understanding for establishing a regimen for drug-resistant cases. Reliable, rapid, and accessible diagnostics that can provide susceptibility information for every candidate anti-TB drug would undoubtedly facilitate the establishment of an appropriate treatment regimen.

Matrix-assisted laser desorption ionization-time of flight mass spectrometry (MALDI-TOF MS) has recently been employed for single nucleic acid polymorphism (SNP) detection, leveraging its simple workflow, short turnaround time, high accuracy, and low cost (Tsai et al., 2012). MALDI-TOF MS utilizes a single-base extension method similar to the Sanger sequencing method, employing dideoxynucleotides (ddNTPs) to extend a single base after the primer. Hence, nucleic acid mass spectrometry is often referred to as a mass spectrometry-based sequencing technique (Hsu et al., 2015). The application of MALDI-TOF MS in pharmacogenomics and the identification of genetic predisposition have validated its reliability and feasibility in SNP detection (Venkatesh et al., 2014; Williams et al., 2020). In 2017, Su et al. (2017) pioneered the detection of drug-resistant TB via the MALDI-TOF MS platform using clinical isolates and specimens. The detection process could be completed within a few hours, with an accuracy comparable to the Sanger sequencing method. In comparison to other detection systems, MALDI-TOF MS acquires the absolute mass value, representing an intrinsic property of a molecule, while others depend on signals of relative electrophoretic mobility or a hybridization event. This disparity enhances the accuracy of the detection outcomes obtained through MALDI-TOF MS.

MassARRAY® (Sequenom, Inc., San Diego, United States) is a MALDI-TOF MS-based method with robust capabilities for detecting drug resistance in tuberculosis (Bouakaze et al., 2011; Su et al., 2017). MassARRAY offers the ability to analyze multiple drug resistance-associated genes in a single reaction, providing the convenience of incorporating additional genes when needed. For instance, the current MassARRAY version covers all 5 first-line anti-TB drugs and more than 10 s-line drugs, including novel anti-TB drugs such as bedaquiline and linezolid (Table 1). However, despite the excitement surrounding this technique, concerns and suspicions have also emerged. The drug resistance detected by MassARRAY in a given patient often cannot be corroborated by another test. This is either because these drugs are not covered by other methods or because the negative results of these tests are due to their less sensitive performance. A few published studies have compared the concordance among MassARRAY, pDST, and DNA sequencing outcomes on strains. Good consistency was achieved for most of the drugs, with some exceptions (Shi et al., 2022; Wu et al., 2022). One study conducted on sputum using MassARRAY and pDST showed good consistency only with rifampin (RIF) and isoniazid (INH) but poor outcomes for kanamycin (KAN) (Wu et al., 2022). These studies could only include culture-positive cases with available pDST outcomes, potentially introducing bias toward patients with a higher acid-fast bacilli (AFB) load in sputum. Furthermore, the low reliability of pDST for TB could lead to misleading outcomes, especially for second-line anti-TB drugs (Xie et al., 2017; Penn-Nicholson et al., 2022). Consequently, relatively lower concordance rates were often observed when using pDST outcomes as a reference compared to DNA sequencing. Therefore, accurately interpreting the resistance outcomes generated by MassARRAY poses a significant challenge.

**Table 1:** Targeted drug-resistant genes and loci of the MassARRAY platform.

Drug*	Targeted gene	Included locus
**Rifampin (RIF)**	*rpoB*	511, 513, 516, 522, 526, 531, 533
**Isoniazid (INH)**	*inhA*	−15
	*katG*	315, 316
Pyrazinamide (PZA)	*pncA*	57
**Ethambutol (EMB)**	*embB*	306, 406
**Fluoroquinolones (FQs)**	*gyrA*	90, 94,
	*gyrB*	538
**Streptomycin (Sm)**	*rpsL*	43, 48
**Amikacin (AK)**	*rrs*	1,401, 1,484
**Kanamycin (Kan)**	*eis*	−15
	*rrs*	1,401, 1,402, 1,408
**Capreomycin (Cm)**	*rrs*	1,401, 1,402, 1,408
**Ethionamide (ETO)** /**protionamide (PTO)**	*inhA*	−15
**Cycloserine (Cs)**	*alr*	261
**p-aminosalicylic acid (PAS)**	*folC*	43
	*thyA*	202, 75
Bedaquiline (BDQ)	*Rv0678*	193, 466
Clofazimine (CFZ)	*Rv0678*	193, 466
Linezolid (LZD)	*rplC*	450

^*^Drug in bold indicating the drug that included in a routine pDST.

In this study, we sought to address this dilemma by comparing the results of MassARRAY with another widely recognized molecular test, the Xpert MTB/RIF assay (Lawn et al., 2013). Our objective was to furnish objective evidence that could aid healthcare providers in comprehending the practical utility of the MALDI-TOF MS technique. Through this, we aimed to empower them to judiciously leverage the outcomes of this technique in clinical practice.

## Materials and methods

### Ethical approval

This study received approval from the Ethics Committees of Henan Chest Hospital (Zhengzhou, China), and written informed consent was obtained from each participant.

### Study design and participants

Participants were consecutively enrolled from July 2021 to November 2022. The patient recruitment criteria were as follows: the patient exhibited symptoms or signs suggestive of TB (World Health Organization, 2013); any of the following tests conducted during this episode of disease yielded positive results, including smear microscopy examination, culture, Xpert assay, or other molecular tests; or all the aforementioned tests yielded negative results, but the patient had a history of TB and was diagnosed with relapse; with a sufficient volume of the clinical specimen; and the patient expressed willingness to undergo MassARRAY testing to detect drug resistance. For pulmonary TB patients, bronchoalveolar lavage fluid (BALF) was the preferred specimen when the patient consented to the invasive bronchoscopic examination. The clinical specimen underwent smear, culture, Xpert assay, and other routine molecular tests for TB diagnosis. Multiple specimens could be tested for the same patient, and various specimen types might be included for patients with involvement of multiple organs. If any of the above assays yielded a positive result, the case was classified as positive with the corresponding test. If the Xpert assay indicated rifampicin resistance, the case was categorized as rifampicin-resistant. A specific specimen was collected separately for MassARRAY testing. Additionally, pDST and species identification were performed once the isolate was successfully recovered.

### Smear and culture

A direct smear was prepared and stained with auramine, then examined using light-emitting diode microscopy. Liquid culture was conducted using the MGIT 960 system (BD Diagnostic Systems, NJ, USA). For all recovered isolates, MPT64 antigen testing (Kaibili Ltd., Hangzhou, China) was employed to confirm the presence of the *Mycobacterium tuberculosis* (Mtb) complex. Non-tuberculous mycobacteria (NTM) were confirmed through molecular testing (Chen et al., 2022).

### Xpert assay

The Xpert assays were conducted following the manufacturer’s instructions. In brief, 1 mL of sputum specimen was processed and detected using the GeneXpert instrument (Cepheid Inc., Sunnyvale, CA, USA).

### Phenotypic drug susceptibility testing

Culture-positive samples underwent pDST using the 96-well microtiter plate assay (manufactured by Zhuhai Yinke Ltd., China). The assay covered 16 drugs, and testing was conducted following the manufacturer’s instructions. The critical concentrations used were based on the guidelines of the Clinical and Laboratory Standards Institute (CLSI, USA) (Gail, 2018).

### MassARRAY testing

All specimens were processed according to a standardized procedure previously outlined by Conlight TB&DR® Detection (Shanghai Conlight Medical Laboratory Co., Ltd., Shanghai, China) (Shi et al., 2022; Wu et al., 2022). The specimens were transported under appropriate cold chain conditions. Subsequently, DNA was extracted from the specimens and used in PCR reactions targeting 15 drug-resistant genes associated with 15 anti-TB drugs. Another extension reaction was conducted with the product from the previous PCR, designed to detect the mutated loci in each targeted gene. Following desalting, the products were spotted onto the chip using the MassARRAY® Nanodispenser, and the chip was inserted into the MassARRAY® Analyzer for detection and analysis.

### Statistical analyses

The sensitivity and specificity of various assays were determined in comparison to the reference standard. The McNemar test was employed to assess the sensitivity and specificity of Mtb or RIF detection between MassARRAY and the Xpert assay. Statistical analysis was conducted using SPSS version 19.0, with differences considered statistically significant at a *p*-value of <0.05.

## Results

### Patient characteristics

**Figure 1:**
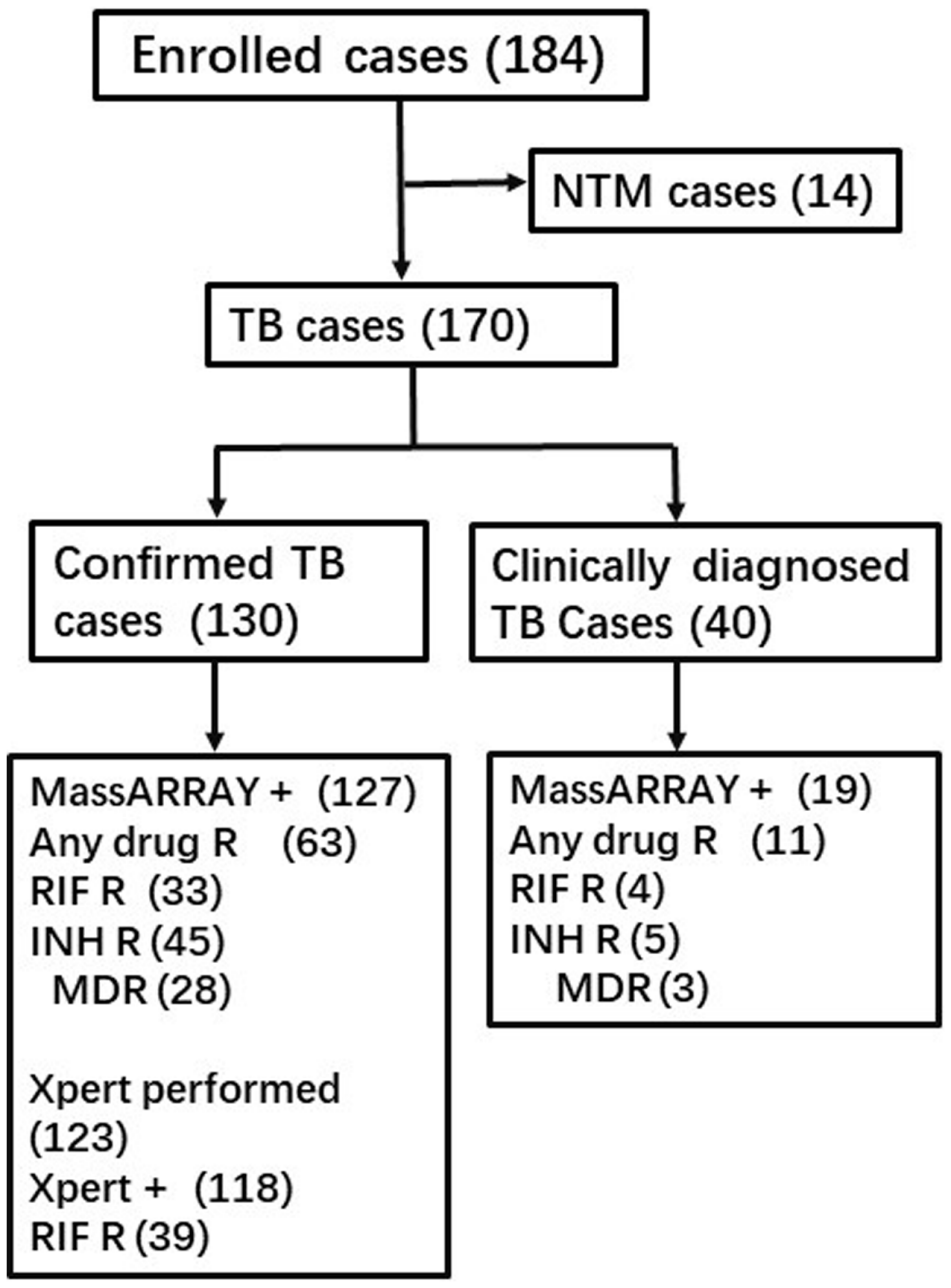
Enrolled patients and the outcomes of MassARRAY and Xpert assay.

A total of 186 participants were enrolled, and 16 of them were subsequently identified as having NTM infections, leading to their exclusion from further analysis. More than one-third of the enrolled cases underwent multiple Xpert assay tests, with a significant number using different specimen types for the repeated assays. Additionally, multiple tests were conducted using smear and culture methods. The reported outcomes of the Xpert assay, smear, and culture hereafter may represent combined results from multiple tests. Among the 170 TB patients, 101 were men, with a median age of 45 years (ranging from 13 to 84). All patients were HIV-negative. The study included 118 pulmonary TB cases, 20 bone and joint TB cases, 15 pleural TB cases, 11 lymph node TB cases, 4 tuberculous meningitis cases, and 2 kidney TB cases. Based on all laboratory testing outcomes except MassARRAY, 130 cases were defined as confirmed TB, while 40 others were clinically diagnosed with TB without any supporting bacterial evidence (Figure 1).

### The overall performance of each diagnostic method in TB diagnosis

Among all the enrolled TB cases, MassARRAY exhibited better sensitivity (85.88%, 146/170) than Xpert assay (76.62%, 118/154), culture (48.91%, 67/137), and smear (25.85%, 38/147) (*p* = 0.032, *p* < 0.001, *p* < 0.001, respectively). In the subset of 130 confirmed TB cases where the diagnosis was established based on the outcomes of different methods, excluding MassARRAY, the sensitivity of MassARRAY was 97.69% (127/130). This was followed by the Xpert assay with a sensitivity of 95.94% (118/123), culture (58.56%, 65/111), and smear (33.23%, 38/111). MassARRAY showed comparable sensitivity to the Xpert assay (*p* = 0.425) but exhibited higher sensitivity than culture and smear testing (*p* < 0.001, *p* < 0.001) (Table 2).

**Table 2:** Performance of different diagnostics in 130 confirmed TB*

TB location	Specimen type (*n*)	MassARRAY+	Smear+	Culture+	Xpert +
Pulmonary	BALF (78)	75/78	22/70	37/66	69/72
	Sputum (20)	20/20	10/17	14/19	18/19
Bone and joint	Pus (14)	14/14	3/12	6/13	13/14
Pleura	Pleural fluid (9)	9/9	2/5	5/6	9/9
Lymph node	Tissue (7)	7/7	1/6	2/5	7/7
Kidney	Urine (2)	2/2	0/1	1/2	2/2
Total specimens		127/130	38/111^&^	65/111^&^	118/123^$^

^*^Denominator indicates the number of specimens performed the corresponding assay, and the numerator indicates the number of specimens yielded positive outcome. ^&^Compared with the MassARRAY, the difference was significant (*p* < 0.001). ^$^Compared with the MassARRAY, the difference was not significant (*p* > 0.05).

### The performance of MassARRAY and Xpert assay in smear-positive cases

A total of 40 patients had positive smear outcomes, and 2 of them were identified as NTM and were subsequently excluded. Both MassARRAY and the Xpert assay detected TB DNA from all the specimens in these 38 cases. Among them, 28 sensitive cases and 9 RIF-resistant cases were uniformly detected by both methods, whereas one case was found to have a *rpoB* mutation by Xpert only but not by MassARRAY, and the outcome from pDST for this case indicated it as RIF-sensitive. The concordance rate between MassARRAY and Xpert assay for *rpoB* mutation detection was 97.37% (37/38).

### The performance of MassARRAY and Xpert assay in culture-positive cases

A total of 75 patients yielded positive culture outcomes, and 8 of them were excluded because of NTM identification, while the Xpert assay was not performed for 3 other cases. Therefore, 64 patients had comparable outcomes. Both MassARRAY and the Xpert assay detected TB DNA from all the specimens in these 64 cases. Among them, 30 were reported to be sensitive to RIF by both methods, whereas 27 had detectable *rpoB* mutations using both methods. Five cases detected *rpoB* mutations by Xpert only, and another two detected *rpoB* mutations by MassARRAY only. Out of the seven cases with discrepant outcomes, only three had pDST results available. Among these, one case showed resistance to RIF and aligned with the Xpert outcome, while the remaining two cases were sensitive to RIF, contradicting the Xpert outcome. The concordance rate between these two tests for *rpoB* mutation detection was 89.06% (57/64).

### MassARRAY outcome among patients with positive Xpert assay outcomes

Out of the 154 patients who underwent the Xpert assay, 118 patients had positive outcomes, but only 102 had determined RIF outcomes. Among these positive cases, 64 were identified as RIF-sensitive by both methods, whereas 31 exhibited the *rpoB* mutation using both methods. Additionally, seven cases were found to have the *rpoB* mutation solely by the Xpert assay. Only two of these seven cases had pDST outcomes, and both were RIF-resistant. The overall concordance rate between these two tests for *rpoB* mutation detection was 93.14% (95/102).

### The head-to-head comparison of different methods in TB diagnosis and detecting RIF resistance

**Figure 2:**
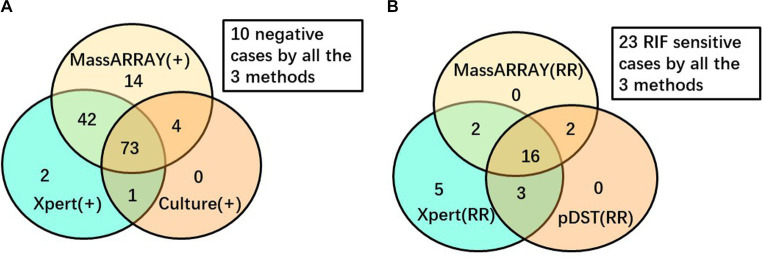
Venn diagram to depict the performance of different diagnostic methods. **(A)** Among 146 cases performed all the 3 tests for TB diagnosis. **(B)** Among 51 cases performed all the 3 tests for RIF resistance detection.

To enhance the objectivity of the comparison, a stratified analysis was conducted (Figure 2). Among the 170 analyzed TB cases, 146 underwent MassARRAY testing, Xpert assay, and culture testing. Out of these, a total of 136 yielded positive results for TB diagnosis. The Venn diagram illustrates that 53.68% (73/136) of these cases were consistently detected by all three methods, while an additional 34.56% (47/136) cases were identified as positive by two of these three methods. In contrast, 14 cases were detected as positive by MALDI-TOF MS only, with 2 and 0 cases detected by Xpert assay or culture only, respectively. Regarding RIF-resistant cases, among the 51 cases that underwent MALDI-TOF MS, Xpert, and pDST for RIF resistance detection, 28 were reported to have RIF resistance by any method. Out of these 28 cases, 16 (57.14%) were consistently detected by all three methods, while another 7 (25%) cases were identified as RIF-resistant by two of these three methods. Additionally, five cases were reported as RIF-resistant by the Xpert assay only.

### The 12 drug resistance detection comparison between MassARRAY and pDST

A total of 33 cases had both MALDI and pDST outcomes, with consistency rates ranging from 78.8 to 93.9% for different drugs (Table 3). Some drugs included in pDST were analyzed as a group due to the over-cross resistance between analog drugs, such as thioacetazone, fluoroquinolone, and second-line injectables. Amikacin exhibited the highest consistency rate at 93.9%, while ethambutol demonstrated the lowest consistency between MassARRAY and pDST.

**Table 3:** Concordance between pDST and MassARRAY in resistance detection.

Drug	MassARRAY	pDST R	pDST S	Concordance rate (%)
RIF	R	16	1	87.9%
	S	3	13	
Isoniazid (INH)	R	17	1	87.9%
	S	3	12	
RIF + INH (MDR)	R	16	1	87.9%
	S	3	13	
Ethambutol	R	6	2	78.8%
	S	5	20	
Fluoroquinolone	R	7	1	87.9%
	S	3	22	
Kanamycin	R	2	2	87.9%
	S	2	27	
Cycloserine	R	0	0	81.8%
	S	6	27	
p-aminosalicylic acid (PAS)	R	0	2	84.8%
	S	3	28	
Streptomycin	R	11	2	87.9%
	S	2	18	
Ethionamide/protionamide	R	1	1	90.0%
	S	2	29	
Amikacin	R	3	1	93.9%
	S	1	28	
Capreomycin	R	2	2	90.9%
	S	1	28	
Second-line injectable reagent	R	3	1	90.9%
	S	2	27	

## Discussion

Tuberculosis necessitates treatment regimens that encompass multiple drugs (World Health Organization, 2022). Therefore, identifying the complete spectrum of drug resistance is a prerequisite for establishing a personalized anti-TB regimen. However, physicians often face challenges in determining an appropriate treatment regimen for the patient due to the absence of drug susceptibility information. While pDST can cover multiple drugs, its value is limited by the fact that outcomes may take months to be obtained. Moreover, growing concerns have been raised about its reproducibility and reliability, as various factors could impact its performance (Yu et al., 2011). Next-generation sequencing (NGS) is a robust platform for detecting mutations in drug resistance-associated genes (Shendure and Ji, 2008). It can simultaneously analyze all genes of interest from clinical specimens and clinical strains within a few days (Goig et al., 2020), and WHO released its guidelines for this promising technique in 2018 (Word Health Organization, 2018). However, due to the expensive equipment required, the complex processing method, low throughput, and the challenging interpretation of sequencing data, NGS is still a few steps away from routine clinical application. MALDI-TOF-MS directly detects the molecular weight of molecules without the need for fluorescent-labeled dye. This feature allows for a higher multiplex of up to 30 targets in one reaction and significantly reduces reagent costs compared to the NGS platform, which requires expensive dye.

WHO recommended the Xpert assay as the initial test for pulmonary TB in 2010 (World Health Organization, 2010). The Xpert assay has demonstrated excellent performance in TB diagnosis and RIF resistance detection (Steingart et al., 2014). Therefore, in this study, we selected the Xpert assay as a reference standard to evaluate the reliability of MassARRAY in TB diagnosis and the detection of RIF resistance. However, for other drugs, the absence of a standardized method, coupled with the limited sensitivity or reproducibility of available pDST or molecular tests, makes it challenging to establish a reliable benchmark for evaluation. Among the enrolled cases, MassARRAY, Xpert assay, and culture all exhibited high reliability, as positive outcomes were observed in 88.24% (120 of 136) of cases that underwent all three tests, and these positive results could be confirmed by at least one of the other two methods. The concordance rate between MassARRAY and the Xpert assay was higher in smear-positive cases than in culture-positive cases, but the difference was not statistically significant (97.37% vs. 89.06%, *p* = 0.259). Smear positivity indicates a high bacilli load in the specimen. The reliability of RIF resistance outcomes in the Xpert assay is associated with the bacilli load, and studies have reported lower reliability of RIF resistance with a “very low” level readout (Wang et al., 2018; Hu et al., 2019). The discrepancy between the MassARRAY and Xpert assay in detecting RIF resistance may mainly be due to the different bacilli loads in different specimens and the heterogeneous nature of resistance. Another advantage of MALDI-TOF MS in mutation detection is that MassARRAY can differentiate heterogeneity of drug resistance genes with higher sensitivity, very independent of the constitution ratio of these sequence types. Among the 28 cases that underwent MassARRAY testing, Xpert, and pDST and demonstrated RIF resistance, 20 cases with RIF-resistant outcomes by MassARRAY were confirmed by at least one of the Xpert assays or pDSTs (Figure 2B). These findings strongly suggest the high reliability of MassARRAY testing in detecting RIF resistance.

In addition to RIF, other drugs detectable by pDST also showed a high level of agreement with the results obtained through MassARRAY analysis, with consistency rates ranging from 81.8 to 93.9%. Furthermore, a concordance rate of 87.9% was achieved for the identification of MDR cases. These concordance rates are generally about 5% lower than those reported by Wu et al. (2022), who conducted their evaluation using clinical isolates. They also obtained the best concordance for kanamycin but the lowest for ethambutol. We hypothesize that the paucibacillary status of the specimens we tested may have influenced the drug detection outcomes in some way (Wang et al., 2018). Many studies focusing on the discrepancy between molecular tests and pDST for drug resistance diagnosis have demonstrated that molecular tests have much better reproducibility than phenotypic DST, meaning the adjusted outcomes after repeated tests favor the molecular test results (Gu et al., 2015; Wu et al., 2022). Hence, we presume that a better authentic performance for MassARRAY could be expected.

Pulmonary TB patients were recommended to collect BALF instead of sputum to enhance the success rate of detection; however, similar positive rates were observed for both groups [63.89% (69 of 108) vs. 66.67% (16 of 24), *p* > 0.05]. This observation may suggest a bias in which patients with higher bacterial loads in sputum are less likely to be advised for an invasive bronchoscopy examination. Further analysis revealed that more than half of the tested sputa were smear-positive, while only 15% showed smear positivity with BALF. This difference might justify considering bronchoscopy examination as a measure for sample collection, particularly for patients with a low AFB load in their sputum. In this study, good performance of MassARRAY was observed for all specimen types except CSF. However, due to the limited number of these extra-pulmonary specimens, it is challenging to draw reliable conclusions.

MALDI-TOF MS is renowned for its high sensitivity in detecting small molecules (Roskey et al., 1996; World Health Organization, 2017). In this study, MassARRAY outperformed the Xpert assay in the etiological diagnosis of TB, even when the Xpert assay outcomes of many patients were a composite result of multiple tests. Su et al. reported that the limit of detection (LOD) of MassARRAY is 5 CFU/mL (Hsu et al., 2015), which is significantly lower than the reported LOD of the Xpert assay (114 CFU/mL) and even lower than the new generation Xpert assay, i.e., Xpert MTB/RIF Ultra (16 CFU/mL) (Chakravorty et al., 2017). More than one-third of the enrolled cases yielded negative outcomes in their initial Xpert tests in this study, while the majority of these cases were successfully detected by MassARRAY. However, based on the principle of MALDI-TOF MS in mutation detection, it may not provide comprehensive coverage for resistant genes lacking mutation hotspots, such as the pyrazinamide (PZA) resistant gene *pncA*. Dozens of *pncA* mutation types related to PZA resistance have been reported, but only one locus of the *pncA* gene was included in the panel of MassARRAY. This limited coverage diminishes the practical value of PZA resistance detection in MassARRAY, so we did not analyze the detection outcome of the *pncA* mutation in this study.

The strength of our study is that we concentrated on the reliability evaluation of MassARRAY against a very stringent reference standard, and our outcomes favor the application of this technique. Although we only extensively analyzed the reliability of RIF resistance detection by MassARRAY, owing to the same rationale for drug resistance detection, the same specimen used, and the same detection reaction performed, we propose that the high reliabilities of other drug resistance detection could be expected. However, as a retrospective study, its limitations should also be noted. First, as the focal control method, the outcomes of the Xpert assay for more than one-third of the enrolled patients were integrations of outcomes of several tests and even on different specimen types in this near episode. Consequently, the performance of the Xpert assay was overstated in this study. Second, we focused on the drug resistance detection capability of MassARRAY; therefore, patients with bacteriological evidence of TB were favored in enrollment, and hence the performance of each involved method for TB diagnosis could not be extrapolated to all TB patients. Third, due to the accessibility of the molecular test, only the Xpert assay was used as a reference in this study. We suggest more comparative studies using MTBDRplus, Xpert MTB/XDR (Cepheid, USA), and other molecular tests as references to better understand the reliability of MALDI-TOF MS in drug resistance detection of other drugs. Finally, the targets of MassARRAY are gene mutations, and their authentic significance in drug resistance indication was not discussed in this study.

In conclusion, MassARRAY was demonstrated as a highly sensitive method in TB diagnostics and a powerful tool for comprehensive TB resistance detection. Given its high consistency in RIF resistance detection compared with the Xpert assay, it is reasonable to speculate that resistant outcomes of other drugs detected by the MassARRAY assay, which often pose difficulty in verification, could be highly predictive of actual drug resistance.

## Data availability statement

The raw data supporting the conclusions of this article will be made available by the authors, without undue reservation.

## Ethics statement

The studies involving humans were approved by Ethics Committees of Henan Chest Hospital (Zhengzhou, China). The studies were conducted in accordance with the local legislation and institutional requirements. The participants provided their written informed consent to participate in this study. Written informed consent was obtained from the individual(s) for the publication of any potentially identifiable images or data included in this article.

## Author contributions

RL: Data curation, Writing – original draft. JL: Data curation, Writing – original draft, Investigation, Methodology. YZ: Methodology, Writing – original draft. HQ: Methodology, Writing – original draft. SB: Writing – original draft, Data curation. FW: Writing – original draft, Methodology. HD: Writing – original draft, Data curation, Writing – review & editing. HH: Data curation, Writing – review & editing, Writing – original draft, Conceptualization, Funding acquisition.
